# Long-term Effects of Remote Patient Monitoring in Patients Living with Diabetes: A Retrospective Look at Participants of the Mississippi Diabetes Telehealth Network Study

**DOI:** 10.1089/tmr.2022.0009

**Published:** 2022-06-28

**Authors:** Tearsanee Carlisle Davis, Ashley S. Allen, Yunxi Zhang

**Affiliations:** ^1^ University of Mississippi Medical, Center for Telehealth, Jackson, Mississippi, USA.; ^2^ Department of Data Science, University of Mississippi Medical Center, John D. Bower School of Population Health, Jackson, Mississippi, USA.

**Keywords:** access, diabetes, remote patient monitoring, rural health, telehealth

## Abstract

**Introduction:**

Remote patient monitoring (RPM) has demonstrated value as a tool to aid patients in management of their chronic illness in the home. Although the Mississippi Diabetes Telehealth Network Study (MSDTNS) was successful in reducing HbgA1c levels for patients participating in RPM in the Mississippi Delta, the long-term effect of RPM on patients and how to support patients to maintain the treatment effect after discharge remain unclear.

**Objective:**

This study evaluated the long-term effectiveness of an RPM program after the intervention was withdrawn.

**Materials and Methods:**

A retrospective review of medical records of patients who completed all phases of the MSDTNS from 2014 to 2016 was performed over a period of 6 months. Data collected included HbgA1c values, demographics, and changes in social determinants of health.

**Results:**

Of the 31 participants, African Americans displayed a significant difference in HbgA1c values compared with Caucasians since the end of the MSDTNS. No significant effect of other variables, such as income, marital status, insurance coverage, or age, on the change in HbgA1c values was detected since the end of the original study.

**Conclusions and Relevance:**

This limited study implies that African Americans are at higher risk for an increase in hemoglobin A1C after the program is completed. More investigation is needed to identify ways to reduce their risk and equalize the long-term effects of RPM on clinical outcomes of patients in rural or underserved communities.

## Introduction

According to the Mississippi State Department of Health, over 13% of Mississippians are living with diabetes, accounting for over 3 billion dollars in annual direct and indirect costs.^1^ The lack of appropriate resources, including but not limited to support and education, hinders patients from effectively managing their diseases.^2^

Telehealth has attracted growing interest as a means of improving patient care and treatment effectiveness. Using remote patient monitoring (RPM), a key component of telehealth, to aid in managing chronic diseases such as diabetes improves clinical and economic outcomes, especially for patients living in rural areas that lack access to health care services.^3^ RPM utilizes innovative digital technologies to closely monitor patient's daily activities, capture accurate clinical information, enhance patient–provider communication for correction of medication administration, and ultimately improve clinical outcomes, such as reducing hospitalizations.^4^

Setting appropriate goals is the initial step in managing diabetes. With the exception of pregnant women, HbgA1c levels should be <7%. Lowering the HbgA1c levels to <7% reduces symptoms of diabetes and its long-term complications such as kidney dysfunction, visual impairment, cerebrovascular events, and heart disease.^2,5^

Identifying barriers to suboptimal glucose control, including lifestyle (diet and exercise), nonadherence to medication/monitoring practices, and lack of knowledge of the disease process, is pertinent in education disbursement and goal development for patients on RPM.^6^ A nurse will then provide individualized diabetes education based on the nurse–patient discussion of mutually set goals.

Mutual goal setting allows the patient to become more engaged in their care, increasing their ability to self-manage their skills related to monitoring their diabetes.^7^ Diabetes management information can be provided to the patient using routine telephone calls, by direct messaging, or during alert situations (notification of elevated glucose) identified by the RPM nurse.^8^

Over the enrollment period, patients learn to appropriately monitor glucose, dietary intake, exercise options, and medication management. The goal is to provide lasting education to carry the patient through their lifetime with the chronic disease state.

Outcomes reported upon completion of the RPM program have typically shown improvements in HbgA1c without showing sustained or at goal HbgA1c levels over time. Other than the expected HbgA1c levels reported, researchers have also investigated reasons behind potential successes, identifying barriers and groups with potential for disparity that would need additional support to improve success rates of RPM for diabetes management.

In a review completed by Andrès et al., RPM was found to show improvements in HbgA1c for participants in addition to reducing instances of hypoglycemia when compared with standard care practices.^6^ In the study led by Michaud et al., RPM for diabetes management was generally found to show improvement in HbgA1c numbers. However, while women were found to be more successful in completion of the study, they had less improvement of HbgA1c at 0.4% when compared with men at 0.8% reduction.^8^

Relatedly, Michaud et al. found that women with public insurance (Medicare and Medicaid), higher baseline HbgA1c values, and reduced engagement in participation had less success with RPM.^9^ Randall et al. found that while enrolled in RPM for diabetes management, patients were attending the same number of in-office primary care visits. This information is attributed to RPM nurses encouraging patients to seek follow-up for trending elevations in glucose, which possibly would not have happened otherwise, thereby mitigating further complications or hospitalizations.^10^

Su et al. found that patients who were more engaged in RPM were more successful in reaching HbgA1c goals in a 6-month time period. They defined better engagement as patients who monitored and uploaded their bio-clinical data more often, usually daily, than those who did not.^7^ Although a multitude of factors may affect the perceived success of RPM in diabetes management, studies by Michaud et al. and Su et al. suggest that patient engagement could be the driving force between marginal and significant improvement of HbgA1c levels.^7,9^

Based on the lack of information regarding follow-up post-RPM completion, more thorough studies should be completed looking at long-term sustainability of HbgA1c levels over a longer duration of time and with larger sample sizes. In their meta-analysis of systemic reviews of randomized controlled trials, Lee et al. recognize the significance that RPM plays in reducing HbgA1c levels, specifically with patients whose HbgA1c level is >8% before implementation.^11^ While RPM has been shown to improve clinical aspects of diabetes such as improved HbgA1c, long-term diabetes maintenance data are lacking and should be explored further.^5^

Recognizing the importance of effective management of chronic diseases for the reduction of overall health care costs, the University of Mississippi Medical Center and its partners initiated the Mississippi Diabetes Telehealth Network Study (MSDTNS) to implement RPM with a collaborative model for managing diabetes.^12^ With the rural health clinic as the center of primary care and the tertiary center as the provider of specialty services, telehealth was used to connect patients living with uncontrolled diabetes to members of the care team not readily available in their communities.

Patients in the study were educated on managing diabetes as well as required to complete the daily health session of measuring their blood glucose at home. The nurses who coached the patients through their experience while on the program then reviewed these measurements. The findings of this program contributed to expansion of the RPM program. Of 171 patients originally enrolled in the MSDTNS, 115 completed all phases of the study.

There was a significant difference in the HgbA1c values from baseline to 3-, 6-, 9-, and 12-month values. Maximum benefit was achieved after 3–4 months on the program and was maintained over the 12-month period. It was concluded that RPM and telehealth could be effective tools for assisting home-based patients in self-management of diabetes in rural areas.

Even with the perceived success of this initial study, the long-term effect of RPM on patients and how to support patients to maintain the treatment effect after discharge remain unclear. To explore this, we conducted a retrospective study on patients who completed all phases of the MSDTNS.

This retrospective study evaluates the long-term effectiveness of an educational intervention and chronic disease management program powered by telehealth after the intervention was withdrawn by comparing participants' most current HbgA1c levels with their levels upon completion of the original study that concluded in 2016. The time from withdrawal of the remote monitoring intervention to attempt to follow-up was ∼4 years.

The research questions were as follows:

1. Were the outcomes from the MSDTNS, specifically the HbgA1c level reductions, sustained after withdrawal of the technological intervention?
2. What is the participant's current financial or social status since completion of the study, which may have affected their health?

The study period for this exploration was 6 months. The original study site served as the site of consent as the providers there had established relationships with the participants. Many of the patients continued care at this site after the original study concluded.

## Materials and Methods

The inclusion criteria for this retrospective study were as follows:

- Completion of the MSDTNS
- 18 Years of age or older
- Not pregnant

Institutional review board approval was secured through the University of Mississippi Medical Center as per policy. Clinic staff who were trained on the research procedures contacted prior study participants by phone and invited them to participate in the follow-up study. Calls were made over a 6-month period in an effort to reach as many participants as possible. After informed consent was obtained, participants were administered a short survey (Supplementary Appendix A1) asking questions related to their current social/economic status that might have affected their health since the end of the MSDTNS.

The responses to the survey were deidentified and coded. The researcher at the site also logged the participants' current HbgA1c values and any results that were available in their electronic health record since the conclusion of the original study. If the participant did not have an HbgA1c test within the past 4 months or was not scheduled for one within the next 3 months, a level was drawn at that time. Other information collected included name, age, race, medical history, marital status, and insurance status. Investigators did not ask questions regarding new health problems since the withdrawal of the remote monitoring program.

Descriptive statistics, mean and standard deviation (SD) for continuous variables and frequency and percentage for categorical variables, were used to summarize end-of-intervention and last HbgA1c measures, the outcome variable, and baseline patient characteristics. The Wilcoxon signed-rank test was conducted to examine the change in HbgA1c from the end of the intervention to follow-up.

Linear regression models were performed on available cases to evaluate the effect of each socioeconomic variable on the change in HbgA1c, adjusting end-of-intervention measures of HbgA1c. If significance was detected in regression at the 0.1 level, we further evaluated the change in HbgA1c for the socioeconomic variable. Then, we conducted the Wilcoxon signed-rank test again to examine the change in HbgA1c for each race group.

In addition, missing values occurred in end-of-intervention HbgA1c, age, and marital status. To maximize the use of available information and avoid bias from missing data, we conducted multiple imputations, a well-established strategy that replaces missing data with several sets of plausible values and aims to provide statistically valid inferences. We conducted 10 imputations using fully conditional specification with the Markov chain Monte Carlo procedure.

Specifically, linear regression is used to impute end-of-intervention HbgA1c and age, and logistic regression is used to impute marital status (married yes/no). All variables are included in the imputation model. Then, a linear regression model was performed on each of the 10 sets to estimate socioeconomic effects on change in HbgA1c, and results were combined using Rubin's rules.^13^

All data analyses were conducted using the SAS statistical software (version 9.4; SAS Institute, Inc., Cary, NC).

## Results

Thirty-one of 115 (27%) completers from the original study consented to participate in this study. The remaining 84 completers were lost to care and could not be found to offer participation in this follow-up study. Basic characteristics of participants are presented in Table 1. There were 21 African Americans and 10 Caucasians, with an average age of 62.87 years (SD = 7.94).

**Table 1.** Basic Characteristics

Continuous variables	*N*	Mean (SD)
Age, years	30	62.87 (7.94)
Baseline HgBA1c	30	7.76 (1.26)
Last HgBA1c	31	8.06 (1.68)

Categorical variables	*N*	Frequency (%)
Race	31	
African American		21 (67.74)
Caucasian		10 (32.26)
Marital status	30	
Married		16 (53.33)
Divorce		3 (10.00)
Single		9 (30.00)
Widowed		2 (6.67)
Medical history	31	
HTN		26 (83.87)
HF		1 (3.23)
Insurance	31	
Blue Cross Blue Shield		12 (38.71)
Ambetter		2 (6.45)
Humana Claims		3 (9.68)
Magnolia Health Plan		4 (12.9)
Medicaid		9 (29.03)
Medicare		14 (45.16)
United Health Care		1 (3.23)
AARP		1 (3.23)
Cigna		1 (3.23)

HF; HTN.

Sixteen participants were married. Among the 26 participants who had hypertension, 1 also had heart failure. Sixteen participants used multiple insurance providers. Medicare was the most frequently used insurance among participants and 14 (45%) participants had it. Thirteen (42%) participants used the Magnolia Health Plan or Medicaid. Other insurance was used by 65% of participants.

The HbgA1c value was recorded at baseline for 30 participants with a mean of 7.76 (SD = 1.26). At the end of the study, the HbgA1c value was increased by 0.27 (SD = 1.77), on average, from the baseline. No significant change was detected at the 0.05 level through the Wilcoxon signed-rank test (*p* = 0.33).

The estimates of linear regression models are displayed in Table 2. Race was slightly associated with the change in HbgA1c (*p* = 0.07). Compared with Caucasians, African Americans are expected to have a higher increase of 1.16 (SD = 0.62, 95% CI −0.11 to 2.42) in the outcome at the end. Other socioeconomic variables do not show a significant effect on the change in HbgA1c.

**Table 2.** Linear Regression Model Estimates

Effects	Estimates (SE)	95% CI	*p*
Age	−0.03 (0.04)	(−0.12 to 0.05)	0.43
Race: African American	1.16 (0.62)	(−0.11 to 2.42)	0.07
Marital status: married	0.04 (0.63)	(−1.26 to 1.33)	0.95
Medical History: HTN or HF	−0.36 (0.82)	(−2.04 to 1.31)	0.66
Insurance: Magnolia Health Plan or Medicaid	−0.13 (0.62)	(−1.40 to 1.13)	0.83
Insurance: Medicare	0.73 (0.60)	(−0.50 to 1.95)	0.23
Insurance: others	−0.37 (0.64)	(−1.68 to 0.95)	0.57

**FIG. 1.**
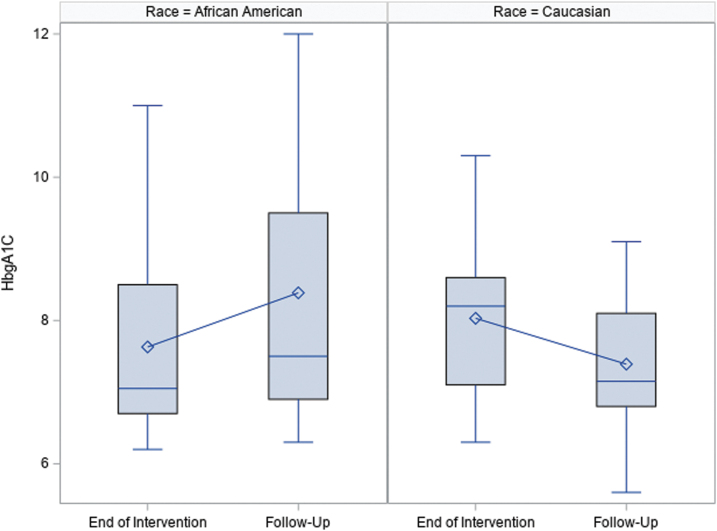
Boxplot HbbA1c by Race.

The change in HbgA1c was further investigated for each race group. African Americans had an average increase of 0.72 (SD = 1.77, *p* = 0.01) in the HbgA1c value, from 7.63 (SD = 1.31) to 8.39 (SD = 1.82), while Caucasians had an average decrease of 0.64 (SD = 1.45, *p* = 0.20) in the HbgA1c value, from 8.03 (SD = 1.18) to 7.39 (SD = 1.15). Figure 1 displays the boxplot of HbgA1c by race.

After multiple imputation on baseline HbgA1c, age, and marital status (married yes/no), results of the regression analysis show that African Americans, on average, have a higher increase of 1.19 (SD = 0.61, 95% CI 0.01 to 2.38, *p* = 0.049) in the change in HbgA1c compared with Caucasians.

## Discussion

The Mississippi Diabetes Network Study completed in North Sunflower County, Mississippi, yielded promising findings that suggested that RPM for chronic disease management could be a viable solution for addressing access to care and education in rural communities. Participants completing that study experienced an average reduction in HbgA1c of 2 points after 12 months of nurse coaching and diabetes self-management education provided through an electronic tablet.^12^

In addition to improved laboratory values, participants lost weight and reported increased knowledge of self-care practices related to diabetes management. Since the conclusion of the study, there has been much discussion regarding the sustained effects once the study ended and patients no longer had access to the nurse coaching.

This investigation aimed to explore the sustained effect of the MSDTNS by assessing the original participants' current HbgA1c levels and performing statistical analyses to determine if there was a significant change after the study period ended. The data from the 31 participants in this follow-up study demonstrate a significant difference in the HbgA1c results of African Americans compared with Caucasians since the end of the MSDTNS. We do not detect a significant effect of other variables, such as income, marital status, insurance coverage, or age, on the change in HbgA1c values since the end of the original study.

This study has a small sample size and missing values with the retrospective nature, which was flawed by the lost follow-up 2 years after the original study completion. This limitation reduces the statistical power of detecting significance. As such, to evaluate the long-term effect of RPM more comprehensively, it would be desirable that future prospective experiments design the study with a follow-up period after removing interventions.

## Conclusions

Providing education and support to patients living with diabetes is vital to ensure a high quality of life with minimal complications. Although there are programs that address the educational needs of patients, many of them require the patient to travel to a certain location at a certain time. For many living in rural underserved areas, this is not possible. RPM for chronic disease management continues to show promise as it is expanded to cover chronic illnesses other than diabetes.

There remains the question of whether patients need additional support after RPM has been withdrawn. The use of technology in health care has proven to be a viable tool to close the gap that exists between need and access. This was proven in the MSDTNS of 2016. The specific aim of that study was to test the ability of an intervention powered by telehealth to aid in the reduction of HbgA1c in patients living in rural Mississippi.

The most recent study aimed to investigate how the former study participants maintained their health status, specifically whether they maintained a healthy HgbA1c level, without any form of monitoring after the conclusion of the MSDTNS. Ninety-seven (84.3%) of the 115 participants who completed the original study were African American and 18 (15.7%) were White.

Although the limited retrospective study does imply that African Americans are at higher risk for having an increase in HbgA1c after the program is completed, more work is needed to investigate the specific reason for this increased risk. Once the reasons for the increased risk are identified, interventions can be tested in this population to reduce their risk and equalize the long-term effects of RPM on clinical outcomes of patients in rural or underserved communities.

Through investigating the actual real-world situation of rural patients, what this study does illuminate is the possibility that RPM programs may prevent patients with chronic conditions from becoming lost to follow-up. While intense RPM programs are not the only solution to prevent gaps in follow-up care, the findings suggest that patients with chronic conditions could benefit from additional support after participating in RPM programs.

## Supplementary Material

Supplemental data

## Supplementary Material

Supplementary Appendix SA1

## Abbreviations Used

HF: Heart Failure
HHS: Health and Human Services
HRSA: Health Resources and Services Administration
HTN: Hypertension
MSDTNS: Mississippi Diabetes Telehealth Network Study
RPM: remote patient monitoring
