# Supplementary material for: Long-term Effects of Remote Patient Monitoring in Patients Living with Diabetes: A Retrospective Look at Participants of the Mississippi Diabetes Telehealth Network Study
Source: Telemed Rep. 2022 Jun 28;3(1):130–6. doi: 10.1089/tmr.2022.0009 (PMC9282779; doi:10.1089/tmr.2022.0009)
Supplement: Supplemental data [file Suppl_AppendixSA1.pdf]

Resize font: ⊞ | ⊟ | Returnin

Retrospective Study Questionnaire

Please complete the survey below.

Thank you!

Please record the patient's Unique Identifier.
* must provide value

1. **Did you have any major changes in employment since October 2016?**
   * must provide value
   - ☐ Lost my job
   - ☐ Got a new job
   - ☐ Hours were reduced
   - ☐ No change

2. **Have you had any changes in your household income since October 2016?**
   * must provide value
   - ☐ Household income increased
   - ☐ Household income decreased
   - ☐ No change

3. **Has there been a change in your marital status since October 2016?**
   * must provide value
   - ☐ Got married
   - ☐ Divorced
   - ☐ Lost a spouse
   - ☐ Separated
   - ☐ No change

4. **Has the number of people in your household changed since October 2016?**
   * must provide value
   - ☐ More people live with me now
   - ☐ Fewer people live with me now
   - ☐ No change

5. **Did you have any insurance changes since October 2016?**
   * must provide value
   - ☐ Lost my insurance
   - ☐ Changed insurance
   - ☐ Was able to get insurance
   - ☐ No change
